# Supplementary material for: Enhanced Oral Absorption of Icaritin by Using Mixed Polymeric Micelles Prepared with a Creative Acid-Base Shift Method
Source: Molecules. 2021 Jun 6;26(11):3450. doi: 10.3390/molecules26113450 (PMC8201319; doi:10.3390/molecules26113450)
Supplement: Supplementary file 1 [file molecules-26-03450-s001.zip › molecules-1234703-supplementary.pdf]

# Supplementary Materials: Enhanced Oral Absorption of Icaritin by Using Mixed Polymeric Micelles Prepared with a Creative Acid-Base Shift Method

**Cheng Tang [1], Xiaoming Chen [2], Hua Yao [2], Haiyan Yin [2], Xiaoping Ma [2], Mingji Jin [2], Xin Lu [2], Quntao Wang [2], Kun Meng [2] and Qipeng Yuan [1,*]**

[1] College of Life Science and Technology, Beijing University of Chemical Technology, Beijing 100029, China; tczy033@126.com

[2] Beijing Shenogen Pharmaceutical Co., Ltd., Beijing 102206, China; xiaoming.chen@shenogen.com (X.C.); hua.yao@shenogen.com (H.Y.); Haiyan.yin@shenogen.com (H.Y.); xiaoping.ma@shenogen.com (X.M.); mingji.jin@shenogen.com (M.J.); xin.lu@shenogen.com (X.L.); quntao.wang@shenogen.com (Q.W.); kun.meng@shenogen.com (K.M.)

* Correspondence: yuanqp@mail.buct.edu.cn; Tel.: +86-10-6443-7610

## 1. Materials

Icaritin (purity ≥ 98.5%) was provided by Beijing Shenogen Pharmaceutical Co. Ltd. (Beijing, China). Cy5 NHS, Cy5.5 NHS, bromomethyl methyl ether (MOMBr), tetrahydrofuran (THF), Dichloromethane (DCM), triethylamine (TEA), Caesium carbonate ($Cs_2CO_3$), Ethyldiisopropylamine (DIPEA), N,N-Dimethylformamide (DMF), isopropanol, and 2-(BOC-amino) ethylebromide were purchased from Beijing Inno-chem Co. Ltd. (Beijing, China).

## 2. Synthesis of Icaritin-Cy5 and Icaritin-Cy5.5

### *2.1. Synthesis Route*

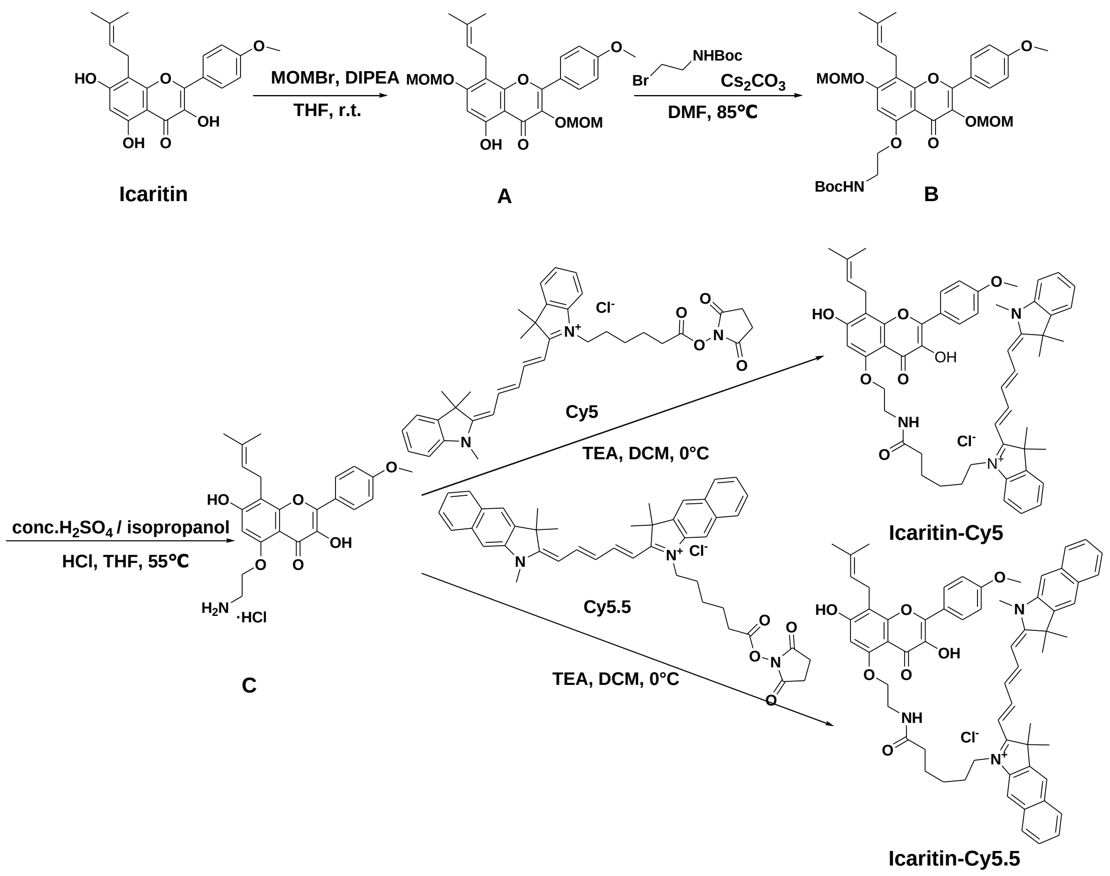


**Figure S1.** Synthesis route of Icaritin-Cy5 and Icaritin-Cy5.5.

### *2.2. Synthesis of Compound A*

**Icaritin** (10.0 g, 27.17 mmol, 1.00 equiv.) and DIPEA (8.06 g, 62.50 mmol, 2.30 equiv.) were dissolved in 100 mL THF and stirred for 30 min at room temperature. The solution was added MOMBr (7.0 g, 55.70 mmol, 2.05 equiv.) dropwise and stirred for one hour. The resulting mixture was filtered, and the filtrate was concentrated under reduced pressure. The crude product was subjected to crystallization by ethyl acetate and petroleum ether to obtain **compound A** (8.32 g, 67%).

$^1$H-NMR (400 MHz, DMSO) δ 12.53 (s, 1H), 8.01 (d, *J* = 8.7 Hz, 2H), 7.13 (d, *J* = 8.8 Hz, 2H), 6.55 (s, 1H), 5.33 (s, 2H), 5.13 (s, 1H), 5.12 (s, 2H), 3.84 (s, 3H), 3.43 (d, *J* = 6.6 Hz, 2H), 3.39 (s, 3H), 3.12 (s, 3H), 1.73 (d, *J* = 14.1 Hz, 3H), 1.61 (s, 3H).$[M]^+$ calcd for $C_{25}H_{29}O_8$, 457.2; found, 456.7.

### *2.3. Synthesis of Compound B*

**Compound A** (5.0 g, 10.96 mmol, 1.00 equiv.), 2-(BOC-amino) ethylebromide (3.67 g, 16.45 mmol, 1.50 equiv.), and $Cs_2CO_3$ (10.70 g, 32.88 mmol, 3.0 equiv.) were added to 100

mL DMF and stirred at room temperature for 30min. The resulting mixture was then heated to 85°C and stirred for 5 h. The solution was quenched with 400 mL water and extracted with 100 mL ethyl acetate. The organic layer was washed with water (5 × 200 mL) and dried over anhydrous sodium sulfate. **Compound B** was obtained by concentration under reduced pressure, and then recrystallized by ethyl acetate and petroleum ether (4.85 g, 74%).

$^{1}$H-NMR (400 MHz, DMSO) δ 7.99 (d, *J* = 9.0 Hz, 2H), 7.12 (d, *J* = 9.0 Hz, 2H), 6.91 (d, *J* = 5.2 Hz, 1H), 6.77 (s, 1H), 5.40 (s, 2H), 5.14 (t, *J* = 5.6 Hz, 1H), 5.09 (s, 2H), 4.07 (s, 2H), 3.84 (s, 3H), 3.46 (dd, *J* = 16.3, 5.0 Hz, 2H), 3.41 (s, 3H), 3.33 (d, *J* = 8.0 Hz, 2H), 3.10 (s, 3H), 1.72 (s, 3H), 1.62 (s, 3H), 1.38 (s, 9H). $[M]^+$ calcd for $C_{32}H_{42}NO_5$, 599.3; found, 599.7.

*2.4. Synthesis of Compound C*

Concentrated sulfuric acid (0.6 g) was added to 15 mL THF and 15 mL isopropanol and stirred for 30 min at room temperature. **Compound B** (1.0 g, 1.70 mmol) was then added to the resulting solution. The solution was heated to 55°C, stirred for 6 hours, and quenched with 30 mL 5% $KHCO_3$ aqueous solution. The resulting mixture was concentrated and filtered. The residue was washed with water and dried at 70°C. The dried residue was dissolved in 40 mL ethanol and 400 μL concentrated hydrochloric acid under refluxing. The resulting solution was then cooled to room temperature slowly and **compound C** crystallized (510 mg, 67%).

$^{1}$H NMR (400 MHz, DMSO) δ 11.03 (s, 1H), 9.08 (s, 1H), 8.35 (s, 2H), 8.11 (d, *J* = 9.0 Hz, 2H), 7.12 (d, *J* = 9.1 Hz, 2H), 6.70 (s, 1H), 5.17 (dd, *J* = 7.4, 6.2 Hz, 1H), 4.27 (t, *J* = 5.2 Hz, 2H), 3.84 (s, 3H), 3.51 – 3.45 (m, 2H), 3.44 (d, *J* = 7.0 Hz, 2H), 1.76 (s, 3H), 1.63 (s, 3H). $[M]^+$ calcd for $C_{23}H_{26}NO_6$, 412.2; found, 411.8. HPLC: purity was 97%.

*2.5. Synthesis of Icaritin-Cy5*

**Compound C** (16.1 mg, 0.036 mmol, 1.1 equiv.), Cy5 NHS(20 mg, 0.033 mmol, 1.0 equiv.), and TEA (20 mg, 0.198 mmol, 6.0 equiv.) were added to 4 mL DCM and stirred at 0°C for 24 hours. The solution was washed with 20 mL water four times and concentrated under reduced pressure to obtain **Icaritin-Cy5** (18 mg, 62%) as a blue powder.

$[M]^+$ calcd for $C_{55}H_{62}N_3O_7$, 876.46; found, 876.3. HPLC: purity was 89.5%.

*2.6. Synthesis of Icaritin-Cy5.5*

**Compound C** (13.8 mg, 0.031 mmol, 1.1 equiv.), Cy5.5 NHS(20 mg, 0.028 mmol, 1.0 equiv.), and TEA (8.5 mg, 0.084 mmol, 3.0 equiv.) were added to 4 mL DCM and stirred at 0°C for 24 hours. The solution was washed with 20 mL water four times and concentrated under reduced pressure to obtain **Icaritin-Cy5.5** (28 mg, 98%) as a green powder.

$[M]^+$ calcd for $C_{63}H_{66}N_3O_7$, 976.49; found, 976.4. HPLC: purity was 95.2%.
